# Room Temperature Ferromagnetic, Anisotropic, Germanium Rich FeGe(001) Alloys

**DOI:** 10.3390/ma6020612

**Published:** 2013-02-21

**Authors:** George A. Lungu, Nicoleta G. Apostol, Laura E. Stoflea, Ruxandra M. Costescu, Dana G. Popescu, Cristian M. Teodorescu

**Affiliations:** National Institute of Materials Physics, Atomistilor 105b, P.O. Box MG-7, Magurele-Ilfov 077125, Romania; E-Mails: adrian.lungu@infim.ro (G.A.L.); nicoleta.gheorghe@infim.ro (N.G.A.); laurastoflea@infim.ro (L.E.S.); ruxandra.costescu@infim.ro (R.M.C.); popescudg@infim.ro (D.G.P.)

**Keywords:** metal-semiconductor compounds, spintronics, surface magnetism, molecular beam epitaxy, X-ray photoelectron spectroscopy, magneto-optical Kerr effect

## Abstract

Ferromagnetic Fe*_x_*Ge_1−*x*_ with *x* = 2%–9% are obtained by Fe deposition onto Ge(001) at high temperatures (500 °C). Low energy electron diffraction (LEED) investigation evidenced the preservation of the (1 × 1) surface structure of Ge(001) with Fe deposition. X-ray photoelectron spectroscopy (XPS) at Ge 3d and Fe 2p core levels evidenced strong Fe diffusion into the Ge substrate and formation of Ge-rich compounds, from FeGe_3_ to approximately FeGe_2_, depending on the amount of Fe deposited. Room temperature magneto-optical Kerr effect (MOKE) evidenced ferromagnetic ordering at room temperature, with about 0.1 Bohr magnetons per Fe atom, and also a clear uniaxial magnetic anisotropy with the in-plane [110] easy magnetization axis. This compound is a good candidate for promising applications in the field of semiconductor spintronics.

## 1. Introduction

Many efforts are concentrated nowadays on systems associating magnetic metals with semiconductors. On one hand, there is a clear need for synthesizing, characterizing and providing recipes for magnetic electrodes in view of spin injection for the emerging field of spintronics [1]. On the other hand, diluted magnetic semiconductors where ferromagnetic ordering is intermediated by indirect exchange represent also an exciting field in view of their possibilities of controlling ferromagnetism via external parameters, such as optically [2,3] or chemically. Summarizing all this consistent work, efforts are dedicated nowadays to synthesize ferromagnetic metals deposited on semiconductors with intermixing and interface reaction as reduced as possible [1,4,5,6,7,8,9]; eventually, if the investigated system is proven to intermix despite efforts such as the use of passivating layers [4,5] or depositing at low temperatures [6,7,10,11], the other possible application is to promote the formation of well defined diluted magnetic systems with structure as well defined as possible and with interesting magnetic properties. In view of this kind of application, systems based on germanium are very promising, since Ge-based systems may be very well interfaced with actual Si-based technology. Secondly, single crystal Ge wavers are cheap and easy to be prepared, *i.e.*, to produce atomically clean surfaces exhibiting long range order.

Following the well-known success of (Ga,Mn)As and (In,Mn)As diluted magnetic semiconductors [12,13], efforts were concentrated also on the synthesis of Mn–Ge based systems [3,14,15]. Comparatively, there are fewer efforts to work on Fe–Ge systems, although iron is a better magnet than manganese. Efforts are continuously concentrated in the synthesis of well ordered and low intermixing Fe layers on Ge(001) [4,16,17,18,19,20,21], (011) [22] or (111) surfaces [23,24]. Basically, most Fe layers are reported to be ferromagnetic with an onset of ferromagnetism at few (3–5) atomic iron layers [19,23]. One phenomenon reported in most of these studies is the occurrence of uniaxial magnetic anisotropy [16,17,18,19,20,21] with the in-plane [110] easy axis for thin Fe films, changing to in-plane easy axis [100] for thicker films [18,19]. The in-plane easy axis [110] can be very well connected to the well known in plane uniaxial magnetic anisotropy of Fe/GaAs(001) [1], whereas the other easy axis is connected to the formation of bulk bcc Fe and eventually to ripples formed on the surface during the growth of the Fe film [18].

However, despite the fact that it is known that a strong interdiffusion of Fe into germanium occurs when the deposition temperature exceeds 400 °C [16], no systematic investigation of systems formed by highly diluted Fe into germanium are reported to date. This was a first stimulus of the actual study: To investigate in which way a system consisting of diluted Fe in Ge(001) may be synthesized and which are its magnetic properties. Indeed, most phases identified to date when Fe is deposited on Ge of various orientations and at various temperatures are Fe-rich phases [22,24], with a single report of a phase with approximate concentration Fe:Ge ≈ 1:2. In the Ge-rich region of the Fe–Ge phase diagram [23], the only stable bulk compound is FeGe_2_, which is antiferromagnetic. A weak ferromagnetic “basal-plane spiral structure” was evidenced between 263 and 298 K for FeGe_2_ by neutron diffraction studies [25]. However, to date no detailed analysis of the magnetic properties of surface Ge-rich phases is reported.

In this paper we will present such an investigation for the first time. By taking into account our previous experience with Fe/Si(001) grown at high temperature [10,11], where a weak but detectable ferromagnetism was reported, we decided to pursue these efforts with growth of Fe on Ge(001). These layers are analyzed *in situ* by low energy electron diffraction (LEED) and by X-ray photoelectron spectroscopy (XPS), together with *ex* *situ* magneto-optical Kerr effect (MOKE) analyses.

## 2. Experimental

The experiments are performed in a surface science complex setup (Specs) composed by a molecular beam epitaxy (MBE), a scanning tunneling microscopy (STM) and a photoelectron spectroscopy chamber. The base pressure in the whole setup is below 2 × 10^−10^ hPa. X-ray photoelectron spectroscopy (XPS) is performed in an analysis chamber is equipped with a 150 mm hemispherical electron energy analyzer (Phoibos), a dual anode (Mg/Al K_α_) X-ray source, a monochromatized (Al K_α_/Ag L_α_) X-ray source and a high power UVS 300 UV lamp. Sample neutralization is achieved by using a flood gun operating at 1 eV electron energy and 100 μA electron current. In this experiment, only monochromatized Al K_α1_ radiation was used (1486.74 eV). The analyzer operated in fixed analyzer transmission (FAT) mode with pass energy of 20 eV; the estimated combined (source + analyzer) resolution is of about 0.75 ± 0.025 eV. The energy was calibrated repeatedly with the Au 4f_7/2_ core level (83.81 eV) using separate thick Au depositions.

**Figure 1:**
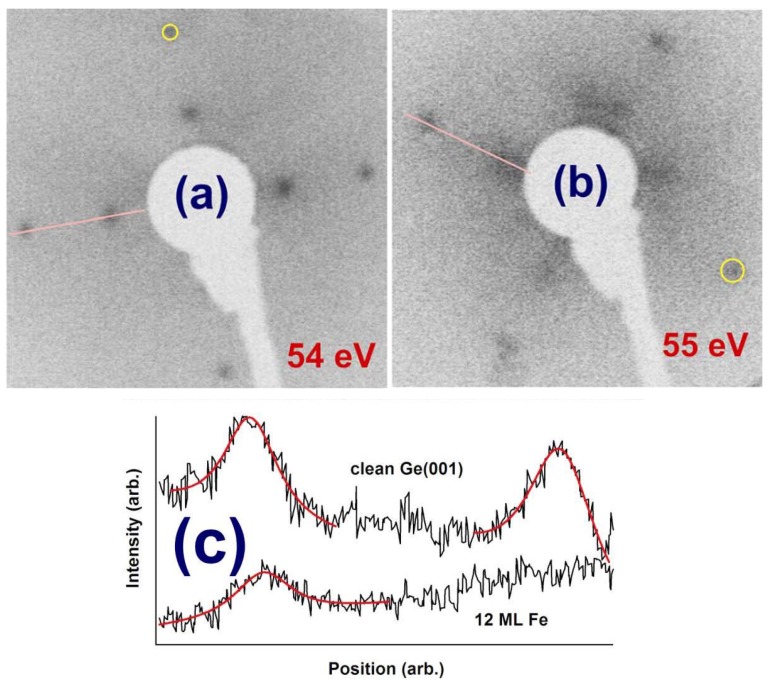
Low energy electron diffraction (LEED) patterns of clean Ge(001) (2 × 1) − (1 × 2) (**a**) and of 12 ML Fe/Ge(001) deposited at 500 °C (**b**). In order to enhance the clarity, negatives of the original photographs are used. The energy used is represented on each pattern. Each pattern has one spot highlighted by a yellow circle, to estimate its approximate broadening; (**c**) LEED spot profile analysis of both images along the designated pink curves in (**a**) and (**b**). Black lines are intensity profiles, red curves are fits using Voigt profiles.

Ge(001) wafers were cleaned by flashing the samples at about 650–700 °C. Clear (2 × 1) − (1 × 2) low energy electron diffraction patterns (LEED) were obtained, as seen in Figure 1. No carbon contamination was detected within the limits of the XPS system (0.05% of a single atomic layer), whereas the oxygen contamination is difficult to be estimated, owing to the superposition of the O 1s peak with the Auger manifold of Ge (LMM, LMV, LVV). Fe was deposited from a properly outgassed Knudsen cell, at a rate of 0.5 Å/min, as calibrated by a quartz thickness monitor. Fe thicknesses are expressed in Fe single atomic layers (ML) with respect to bulk *bcc* Fe (1 ML ≈ 1.433 Å). Again, no carbon contamination was observed. During the deposition, the substrates were held at 500 °C. Lower temperature depositions resulted in layers without any LEED pattern. In choosing the deposition temperature, we took also advantage of a preliminary experiment on Fe/Si(001) [10,11], where a LEED pattern was obtained only for deposition temperatures on the order of 500 °C.

After preparation, Fe/Ge(001) samples were immediately covered by 3 nm of Au, checked again by XPS for confirming the energy calibration and that there is no intermixing between the sample and the capping layer, and removed from the UHV system for *ex situ* analysis by magneto-optical Kerr effect. Longitudinal MOKE was performed at room temperature by using a AMACC Anderberg and Modéer Accelerator AB system, which allows a maximum applied field of 0.6 T. In order to enhance the precision of measurement in the low field region, a maximum field of 0.1 T was applied in the actual experiments.

## 3. Results and Discussion

### 3.1. Low Energy Electron Diffraction

**Figure 2:**
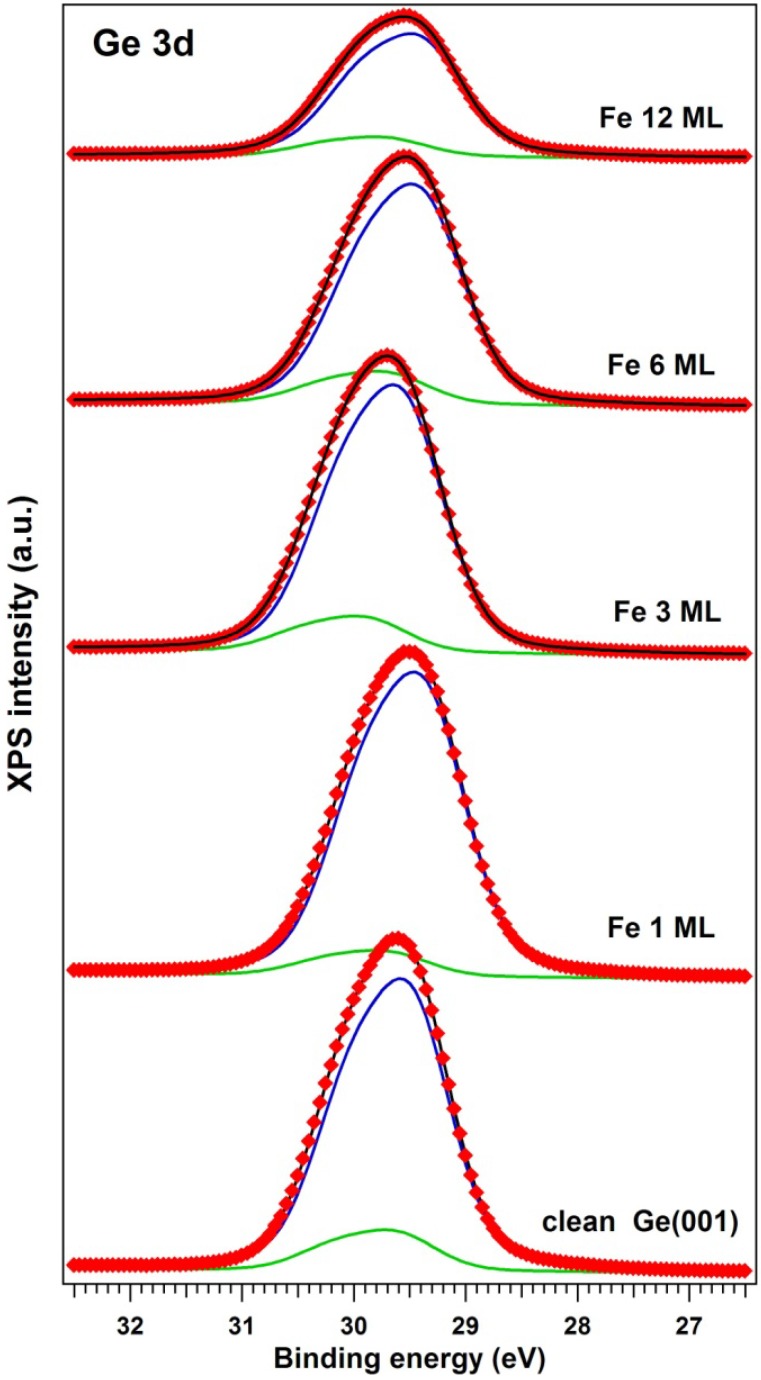
Ge 3d electron distribution curves (EDCs) for clean Ge(001) and for Fe/Ge(001) deposited at 500 °C. The experimental data (red markers) are simulated with a fit with two spin-orbit split Voigt doublets. The separate components are the blue and the green curve; the total fitting function is the black curve.

Figure 1 presents LEED images obtained at 54–55 eV electron energies on the clean Ge(001) sample and on 12 ML Fe deposited on Ge(001). Visible LEED patterns were obtained on the sample with 12 ML Fe also at energies of 47, 68 and 88 eV, but no patterns were visible at higher electron energies, whereas the clean Ge(001) exhibits a rich LEED structure up to about 200 eV with the present setup. Therefore, the surface is disordered by Fe deposition. The (1 × 2) − (2 × 1) reconstruction practically disappears and in fact the only relevant (1 × 1) LEED pattern is the one represented in Figure 2b. Note also that 1 ML Fe/Ge(001) exhibited a clear LEED pattern, with (1 × 2) − (2 × 1) spots; starting with 3 ML the LEED spots became barely visible, then the (1 × 1) spots appear again for the thickest layer investigated in this series, 12 ML.

However, this is an encouraging result pointing on the survival of the long range crystalline order on the FeGe(001) surface. Generally, few LEED patterns are obtained for metals deposited on semiconductors. In [10], Fe/Si(001) exhibited a LEED pattern only up to about 0.8 nm of Fe deposited, whereas in [26,27] Sm/Si(001) deposited at 300 °C showed a very diffuse LEED pattern for 3.2 nm of Sm deposited. Here, we obtained clear LEED patterns for the equivalent of 1.7 nm Fe deposited. (All thicknesses are referenced to the crystal structure of the metal deposited.)

Moreover, although from a first inspection of Figure 1a,b it seems that the spots are broader on the FeGe(001) surface, in fact, a spot profile analysis, represented in Figure 1c yielded results not far away from that of the clean Ge(001) surface. More specifically, the profiles were fit with a Voigt profile [28], which is a convolution between Lorentz and Gauss profiles. The Gauss profile accounts for the spatial distribution of the electron spot and for thermal broadening, whereas the Lorentz profile is connected to the coherence length, as usual in diffraction. The Gaussian broadening was similar in both cases (by about 2% lower for the FeGe(001) film, which may be attributed to a better focusing of the electron beam in the latter case). The Lorentz full width at half maximum (FWHM), divided by the size of the two-dimensional Brillouin zone *q*_0_, is connected to the coherence domain size in the direct space *D* (which will be understood in the following as a typical lateral size of crystalline ordered domains) via the following equation Δ*q*/*q*_0_ ≈ *a*/*D*, where *a* is the surface lattice constant, about 4.0 Å for Ge(001). The obtained normalized Lorentz FWHM is Δ*q*/*q*_0_ ≈ 0.086 for clean Ge(001) and 0.095 for FeGe(001). From this normalized Lorentz width one has to subtract the transfer width of the setup, of about 0.01 [26]. The net result is a coherence length *D* of about 5.2 nm for clean Ge(001), which decreases to about 4.6 nm for FeGe(001). Therefore, the crystalline parts of the surface are not that affected by the presence of Fe.

### 3.2. X-ray Photoelectron Spectroscopy

**Figure 3:**
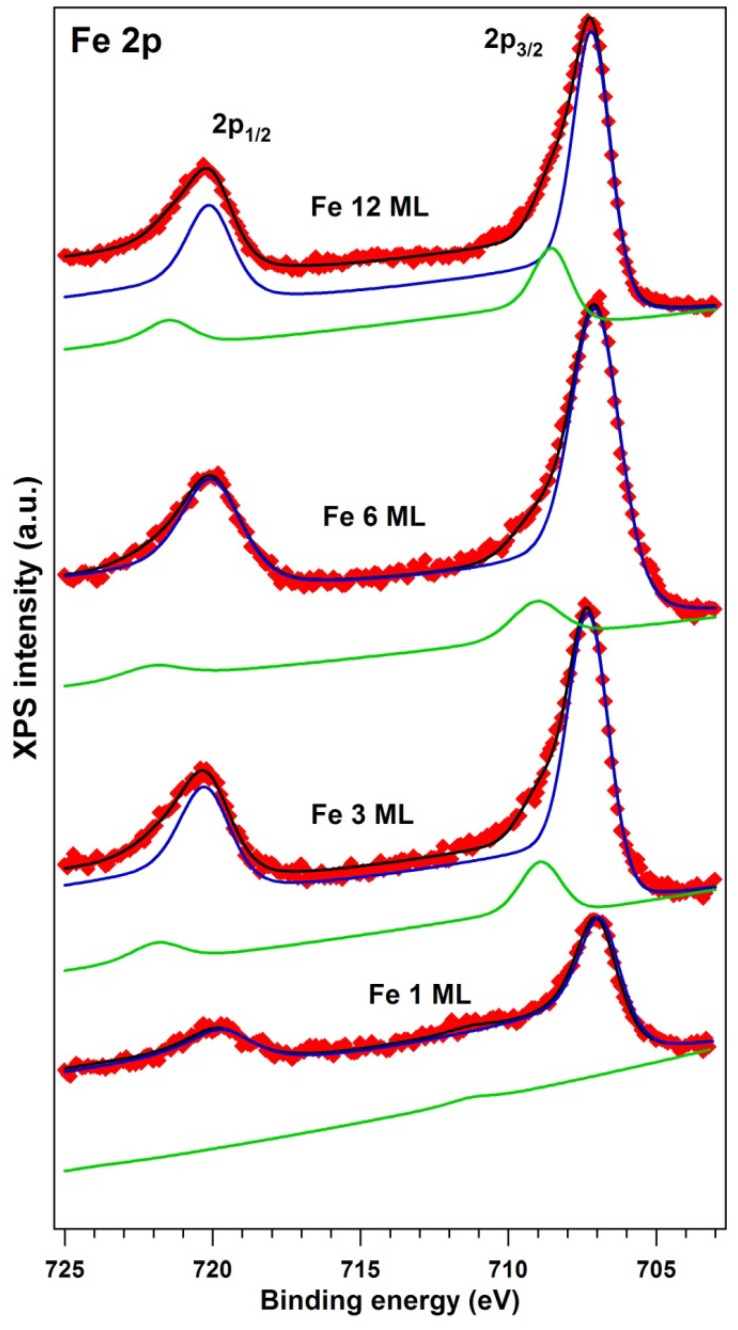
Fe 2p EDCs for all Fe depositions on Ge(001). Same comments as for Figure 2 are applicable. This time the spin-orbit splitting is quite visible between the 2p_3/2_ and 2p_1/2_ components.

The X-ray photoelectron spectra (XPS) are represented in Figure 2 for Ge 3d core levels and in Figure 3 for Fe 2p core levels. The XPS data analysis was performed by simulations using Voigt lines and Voigt inelastic backgrounds [28]. The results of these “deconvolutions” are summarized in Table 1. All the other parameters obtained from fit were in good agreement with previous work: The branching ratios were fixed to their theoretical values (1.5 for Ge 3d and 2 for Fe 2p); allowing variation of these parameters did not improve the quality of the fits. The spin-orbit splittings obtained for Ge 3d were 0.585 ± 0.003 eV, in good agreement with [29] and with our previous work [3]. The Fe 2p spin-orbit splitting yielded 12.95 ± 0.03 eV. Fe 2p has also considerable inelastic background ratios, pointing on the bulk origin of both Fe components [30].

The second Fe 2p component, Fe(2), at higher binding energies, might eventually be due to some contamination of the Fe layer. Its energy (708.5–709.0 eV) is close to some old reports on Fe_3_O_4_ or on oxygen adsorbed on Fe layers, according to the NIST XPS database. In [22] also, 709 eV was reported for surface Fe–O. Since we did not identify carbon or oxygen in the XPS spectra, we cannot immediately assume the presence of iron oxide on the surface; however, in the following we will be rather pessimistic and exclude this smaller, higher binding energy Fe component from the analysis.

The Ge 3d spectra could be simulated with only two components. For clean Ge, these components correspond to surface atoms (the higher binding energy component) and bulk atoms (the lower binding energy component, of higher intensity). Subsurface atoms and an eventual different signature of higher or lower atoms from the buckled dimmers [31] are not clearly identifiable within the resolution of the actual experiment. In the following, we will constantly attribute the component at 29.4 ± 0.1 eV to bulk germanium. With Fe deposition, it seems that an important part of the Ge 3d signal is concentrated in this lower binding energy component. Also, its amplitude decreases constantly with Fe deposition. Therefore, we suggest that this component represents Ge atoms not affected by the presence of Fe.

In Table 1, the integral amplitudes obtained by fit were already corrected by the XPS atomic sensitivity factors [32] of 0.38 for Ge 3d and 3.0 for Fe 2p.

A first evaluation may be done by dividing the Fe(1) main component, with binding energy close to that of metal Fe (about 707 eV) to the total Ge signal. One obtains the ratios from the third column from the right hand side of Table 1. This implies that Fe is embedded in germanium with percentages ranging from 2.4 to 8.6 at %.

A second evaluation may be done by dividing the Fe(1) signal to the second component of germanium, Ge(2), which should represent germanium that reacted with Fe. It may be surprising to attribute a higher binding energy component to Ge reacted with Fe, since usually semiconductors reacting with metals develop lower binding energy components [26,27]; however, for Fe–Ge, a similar binding energy (29.8 eV) was reported in [22]. The Fe(1):Ge(2) ratios represented in the next to the last column in Table 1 suggests the presence of surface compounds of approximate compositions ranging from FeGe_3_ to Fe_3_Ge_5_, as indicated in the last column of Table 1. As mentioned in the Introduction, the Fe–Ge phase diagram [23] does not show too many stable Ge-rich stable compounds. The only stable compound is represented by FeGe_2_. This is a layered compound of (C16) structure, antiferromagnetic at low temperature and with a spiral spin configuration at temperatures between 263 and 289 K [25]. However, as will be discussed in the following paragraph, our samples exhibited a clear ferromagnetic behavior at room temperature (298 K). This is again proof that magnetism at interfaces may differ significantly from the bulk, owing to symmetry breaking or surface charge depletion [1,7].

**Table 1:** Results obtained from the simulation of XPS spectra. Amplitude columns are integral amplitudes, expressed in 10^3^ counts/s × eV ≡ kcps × eV (abbreviated kV), normalized to integral amplitude atomic sensitivity factors (0.38 for Ge 3d, 3 for Fe 2p) [32]. Errors are ±0.005 eV for energies, ±10% for amplitudes (uncertainities are mainly from the atomic sensitivity factors). The last three columns represent amplitude ratio of the lower binding energy of Fe [Fe(1)] to the total Ge signal [Ge(1) + Ge(2)], and the lower binding energy Fe [Fe(1)] divided by the “reacted” Ge component [Ge(2)]. The last column represents a proposed interface compound based on the [Fe(1)]/[Ge(2)] ratio. Italics represent values of lower confidence (see text for details).

Level θ(Å)	Ge 3d (1)		Ge 3d (2)		Fe 2p (1)		Fe 2p (2)		Fe(1):Ge_tot_	Fe(1):Ge(2)	Compound
	E(eV)	A(kV)	E(eV)	A(kV)	E(eV)	A(kV)	E(eV)	A(kV)			
0	29.46	82.5	29.59	11.8	–	–	–	–	–	–	Ge
1	29.33	88.3	29.67	7.6	706.98	2.3	711.25	0.08	0.024	0.305	FeGe_3_
3	29.52	76.5	29.85	10.5	707.27	4.1	708.88	0.80	0.047	0.392	Fe_2_Ge_5_
6	29.35	63.3	29.66	9.5	707.03	5.6	709.05	0.73	0.077	0.590	Fe_3_Ge_5_
12	29.37	37.8	29.68	6.3	707.16	3.8	708.49	0.99	0.086	0.603	Fe_3_Ge_5_

The strongest conclusion from the XPS analysis is related to the Ge-rich character of the interface. One might argue that electron inelastic mean free path (IMFP) effects on the order of 1 nm for Fe 2p electrons and of 2 nm for Ge 3d electrons [33] have to be taken into account; however, a detailed analysis reveals the fact that for the thickest layer investigated in this study, its total thickness is on the order of 3 *λ*_αωγ_, where *λ*_avg_ is the average IMFP, about 1.5 nm. Moreover, working only with the “reacted” Ge 3d component ensures that the composition estimates are related only to the Fe–Ge reacted layer. It may happen that the composition varies with the depth from the sample surface; therefore, all estimates from the last two columns of Table 1 may be regarded just as averages on the investigated thickness of 4–5 nm.

### 3.3. Magneto-Optical Kerr Effect

**Figure 4:**
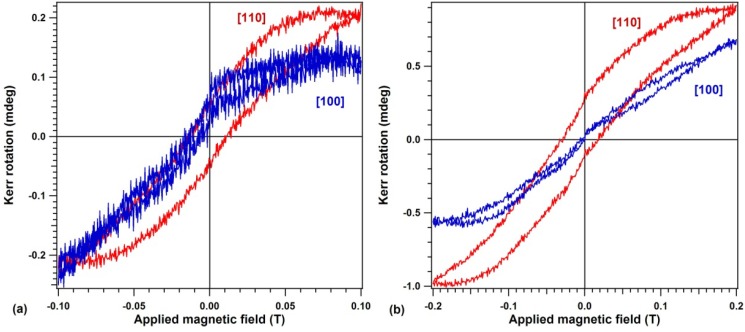
Magneto-optical (MOKE) hysteresis loop for a sample consisting of 12 ML Fe deposited in Ge(001) at 500 °C, with the linear polarization vector of the incident light in the plane defined by the [001] and [100] direction (blue curve), and in the plane defined by the [001] and [110] direction (red curve). (**a**) Maximum applied field 0.1 T; (**b**) Maximum applied field 0.2 T.

Figure 4 represents magneto-optical Kerr effect (MOKE) hysteresis loops obtained on the capped 12 ML FeGe(001) sample with the linear polarization of the light contained in the plane defined either by the [001] off-normal direction with one in-plane [100] direction (or, equivalently, with the magnetic field vector oriented along the [101] direction), or with the sample rotated by 45° along its surface normal [001], *i.e.*, with the light linear polarization contained in the plane defined by [001] and one in-plane [110] direction, equivalent to magnetic field oriented along [111]. A different behavior is observed along these directions. It seems that the [110] axis behaves like an easy magnetization axis, whereas the [100] axis behaves as a hard magnetization axes. This observation is in line with most reports on the uniaxial magnetic anisotropy in Fe layers grown on Ge in different conditions [16,17,18,19,20,21]. Nevertheless, this is the first time that such a phenomenon is observed for a so diluted Fe system in Ge(001). For the origin of the magnetic anisotropy, one cannot invoke in this case preparation conditions, such as e.g., oblique evaporation, since the evaporation proceeded here almost normal to the surface and, anyway, the substrate temperature washes out any initial momentum of the incoming atoms. We rather prefer to infer that this uniaxial magnetic anisotropy is an intrinsic property of magnetic metals accommodating either into, or on, diamond-like (or zincblende) structures. If, for instance, we try to simulate a hypothetical Fe_3_Ge_5_ (or even a Fe_2_Ge_6_) compound as starting from a zincblende Fe_4_Ge_4_ cell and substituting one Fe atom by one Ge, there will still be a high probability to find a second order Fe neighbor for a Fe atom. The line connecting these two neighbors belongs to the {110} family of lines. It is then straightforward that an eventual arrangement of the momenta is favored when the orienting field is applied along one of these directions.

The total MOKE signal obtained from Figure 4b of about 1 mdeg may be used to estimate the magnetic moment of Fe, together with the XPS data. For the calibration of the present setup [26], 2.5 mdeg of MOKE signal corresponds to 1 nm of a bulk bcc Fe layer with a density of 0.85 × 10^23^ cm^−3^) and with 2.2 μ_B_ per atom. In the actual sample there are about 1.7 nm of Fe deposited, computed with respect to the bulk *bcc* Fe density. Therefore, the MOKE signal implies an atomic magnetic moment of (1/2.5) × (1/1.7) × 2.2 μ_B_ ≈ 0.75 μ_B_ per Fe atom. A similar computation yields about 0.1 μ_B_ per Fe atom for the lower field hysteresis curves from Figure 4a. Therefore, the maximum magnetic moment observed in this experiment is about one third from the signal of the bulk Fe at room temperature. Small Fe moments were reported also in other cases for Fe atoms located at the interface with Ge [34]. On the other hand, although measurements at larger fields were not performed during this series of experiments, there are serious reasons to believe that the hysteresis loop from Figure 4b is still a minor loop and the maximum Fe moment approaches about 1 μ_B_, which seems to be a universally accepted value for the atomic magnetic moment measured at room temperature on reacted Fe layers deposited on III–V semiconductors [6,7,9]. We must stress here that this is the first time that ferromagnetism is detected in a Ge-rich Fe–Ge surface compound. Also, along the [110] in-plane direction the ferromagnetism is quite robust, since the coercitive field is about 230 Oe, much larger than the reported coercitive fields for Fe grown on Ge [16,17,18,19,20,21]. The measured coercitive fields for FeGe(001) largely exceed that of metal Fe or even of reacted Fe on Si(001) [10,11]. There seems to be also a small exchange bias pf about 50 Oe in Figure 4b, since for the measurement with maximum applied field 0.2 T the two coercitive fields are 180 Oe in the positive direction and 280 Oe in the negative direction. In the absence of more detailed investigations, we cannot yet suggest the origin of the eventual two phases (ferromagnetic and antiferromagnetic) present at the sample’s surface. It is tempting to assess the overall composition derived in Table 1 for the 12 ML sample as Fe_3_Ge_5_ being composed of FeGe_2_ (antiferromagnetic) + FeGe (ferromagnetic). If this were true, then Fe in the ferromagnetic phase would have an atomic magnetic moment close to that of the bulk Fe, since only one third of the Fe atoms would be ferromagnetic ordered. However, in absence of more detailed determinations by high resolution XPS using synchrotron radiation and/or photoelectron diffraction (planned for the future), one cannot be completely sure about this hypothesis. Also, future measurements on temperature dependence of magnetization will be investigated to elucidate this aspect.

**Figure 5:**
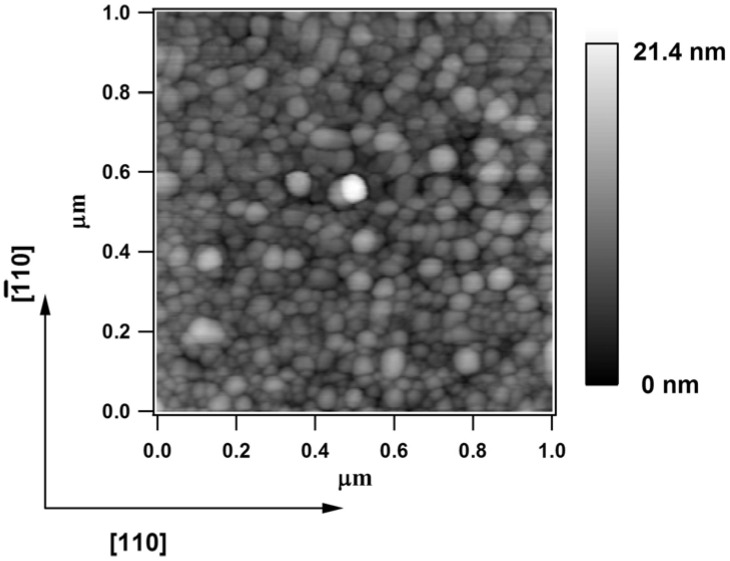
Atomic force microscopy images obtained at room temperature and in air on 12 ML Fe deposited on Ge(001) at 500 °C, amplitude signal.

In the case of Fe deposited on Si(001) at similar elevated temperatures (470 °C), Reference [35] reported AFM images exhibiting elongated islands of approximate size (10 × 20) to (10 × 50) nm^2^, with the long edge aligned along the [110] directions. Such elongated islands might be the origin of the observed easier magnetization axis along [110]. In order to check this hypothesis, we performed a similar AFM investigation under air (Asylum research, at room temperature), represented in Figure 5. It can easily be seen that the sample surface is nanostructured, with the formation of regular islands of approximate size of 20–30 nm. We did not observe the formation of elongated islands, as exhibited by Figure 2 of [35], although several scans were performed. The surface looks quite homogenous and Figure 5 represents a typical example of a surface topology. The computed root mean square (RMS) roughness is 2.34 nm, which is slightly larger than the equivalent thickness of the deposited Fe film (12 ML ≈ 1.72 nm), but considerably lower than the equivalent thickness of a diamondlike FeGe(001) structure obtained with the same amount of Fe deposited: the density of Fe atoms of such a structure is about four times lower than in bulk *bcc* Fe, therefore the equivalent thickness of FeGe formed at the surface would be in the range of 6.88 nm. We recall that, anyway, the absence of elongated Fe-containing stripes excludes the hypothesis that some shape anisotropy is responsible for the observed anisotropy in the MOKE hysteresis loops. We conclude by proposing that the observed anisotropy is a true microscopic property, not connected to the surface morphology.

Finally, the small displaced loops obtained with the field along the [100] direction for a maximum applied field of 0.2 T (Figure 4b) are not a sign of ferromagnetism; they were recorded also for a separate sample consisting on 3 nm Cu deposited on Ge(001), used to test the MOKE system for any spurious signal. Therefore, one may infer that the area of the hysteresis loop when the field is applied along [100] is almost zero.

## 4. Conclusions

This paper presented the first evidence of a long range ordered interface (exhibiting clear LEED patterns) formed by Fe highly diluted in Ge(001), exhibiting ferromagnetism and uniaxial magnetic anisotropy. XPS analysis revealed that the concentration of Fe in Ge (at least within the depth sensitivity of the XPS method, of about 4–5 nm [33]) is of 2%–9%. By associating the relevant Fe 2p signal with only one of the Ge 3d components, attributed to Ge reacting with Fe, an approximate composition of FeGe_2_ or, more precisely Fe_3_Ge_5_, is derived. It is suggested that the uniaxial magnetic anisotropy is rather an intrinsic property occurring each time magnetic ions are related somehow (embedded or deposited on) diamond-like or zincblende structures. The maximum Fe magnetic moment measured at room temperature is on the order of 0.75 μ_B_, and is expected to increase by lowering the temperature or applying larger fields. These variable temperature experiments are planned for the future, together with high resolution transmission electron microscopy analyses. Also, the detected exchange bias suggests the co-existence of a ferromagnetic phase with an antiferromagnetic one, possibly FeGe and FeGe_2._ High resolution XPS and photoelectron diffraction experiments are planned to clarify these aspects.
